# Toscana Virus in Spain

**DOI:** 10.3201/eid1111.050851

**Published:** 2005-11

**Authors:** Sara Sanbonmatsu-Gámez, Mercedes Pérez-Ruiz, Ximena Collao, María Paz Sánchez-Seco, Francisco Morillas-Márquez, Manuel de la Rosa-Fraile, José María Navarro-Marí, Antonio Tenorio

**Affiliations:** * Hospital Universitario Virgen de las Nieves, Granada, Spain; † Instituto de Salud Carlos III, Madrid, Spain; ‡ Universidad de Granada, Granada, Spain

**Keywords:** Toscana virus, seroprevalence, phylogeny, vectors, Spain, research

## Abstract

Toscana virus (TOSV, *Phlebovirus*, family *Bunyaviridae*) infection is one of the most prevalent arboviruses in Spain. Within the objectives of a multidisciplinary network, a study on the epidemiology of TOSV was conducted in Granada, in southern Spain. The overall seroprevalence rate was 24.9%, significantly increasing with age. TOSV was detected in 3 of 103 sandfly pools by viral culture or reverse transcription–polymerase chain reaction from a region of the L gene. Nucleotide sequence homology was 99%–100% in TOSV from vectors and patients and 80%–81% compared to the Italian strain ISS Phl.3. Sequencing of the N gene of TOSV isolates from patients and vectors indicated 87%–88% and 100% homology at the nucleotide and amino acid levels, respectively, compared to the Italian strain. These findings demonstrate the circulation of at least 2 different lineages of TOSV in the Mediterranean basin, the Italian lineage and the Spanish lineage.

## Abstract 2

At least 2 virus lineages are circulating in the Mediterranean basin.

Within the last decade, the emergence and reemergence of arthropodborne virus (arbovirus) infections has been a health problem worldwide. West Nile virus (WNV) infection is a seasonal epidemic in North America (*1*). In southern Europe, WNV infections (*2**–**4*), tickborne encephalitis (*5*), sandfly fever Sicilian virus (SFSV), sandfly fever Naples virus (SFNV), and Toscana virus (TOSV) infections have been reported in Mediterranean countries (*6**,**7*). In Spain, a multidisciplinary network, EVITAR, has been recently created to study arthropod- and rodentborne viral diseases. One of the objectives of the network is to study TOSV infections in Spain.

TOSV (genus *Phlebovirus*, family *Bunyaviridae*) is an important agent of acute meningitis and meningoencephalitis in residents and visitors from Mediterranean countries (*7**–**13*). Aside from TOSV, other sandfly fever viruses, i.e., SFSV and SFNV, cause a brief, self-limiting febrile illness (*6*). Although TOSV is not normally associated with mild disease, serologic studies report high seroprevalence rates in areas of confirmed TOSV infections (*7**,**14**,**15*). Furthermore, a case of influenzalike illness caused by TOSV has recently been reported (*16*). In Spain, the first TOSV infections involving the central nervous system were reported in Granada in 1988 (*7*). Later, cases of TOSV infections were detected in other areas of Spain (*15*). Phylogenetic analysis of short polymerase chain reaction (PCR) products from the L segment showed that nucleotide sequences of TOSV isolates from Granada differ significantly from the Italian strain ISS Phl.3 (*17*).

TOSV was first isolated in Italy from the sandfly *Phlebotomus perniciosus* and later from *P. perfiliewi* (*8**,**18*). *P. perniciosus* is the most abundant anthropophilic species of *Phlebotomus* in Spain (*19*). The maximum activity of sandfly vectors for TOSV occurs during summer, along with most cases of TOSV infection (*7*). Vector-based TOSV surveillance is useful in reporting virus activity. It provides predictive indicators of transmission activity level associated with elevated human risk. However, no data are available on detection of TOSV from vectors in Spain.

As part of the study of TOSV infection within the aims of the EVITAR network, this work focused on 3 main objectives. First, a seroprevalence study to detect TOSV immunoglobulin G (IgG) antibodies was conducted. Second, by means of viral culture and reverse transcription (RT)-PCR, we investigated the presence of TOSV in pools of phlebotomine sandflies. Finally, positive pools and viral isolates were phylogenetically characterized.

## Materials and Methods

### Prevalence Study of Anti-TOSV IgG Antibodies

#### Population Study for Selecting Participants

**Figure 1:**
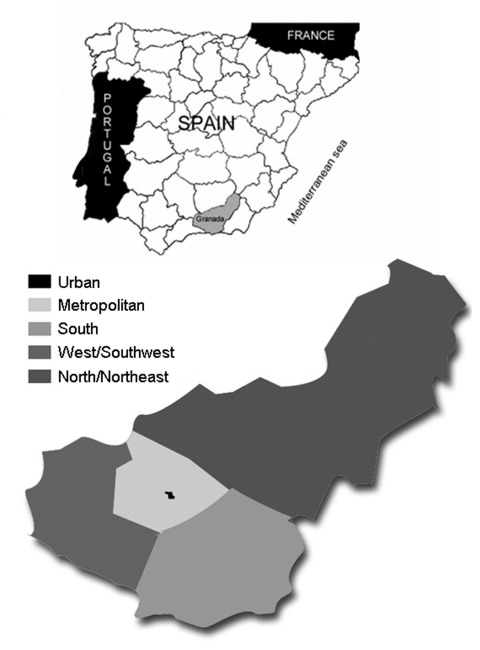
Upper map, geographic situation of the study population (Granada province) in Spain; lower, distribution of geographic areas in Granada province for the seroprevalence study of anti–Toscana virus immunoglobulin G antibodies.

The seroprevalence study was conducted on study participants from the Granada population. Participants were retrospectively selected on the basis of demographic data and estimations of seroprevalence rates to TOSV (*7**,**15*). To evaluate differences in seroprevalence rates within Granada, the province was divided into 5 geographic areas: urban, metropolitan, south, west/southwest, and north/northeast (Figure 1). By age groups, 20% were <18 years, 65% were 18–65 years, and 15% were >65 years of age.

#### Serum Samples

Serum samples were collected from September to December 2003. Specimens from adults 18- to 65-years of age were collected from anonymous healthy blood donors. Specimens from persons <18 and >65 years of age were obtained from 2 laboratories in Granada from persons with noninfectious pathologic features. Only data on age, sex, and geographic area of origin were recorded from the study population. Anti-TOSV IgG was detected by a commercial enzymatic immunoassay, EIA Enzywell Toscana virus IgG (Diesse, Italy), following the manufacturer's instructions.

### Investigation of TOSV in Vectors

#### Capture of Phlebotomine Sandflies

Phlebotomine sandflies were captured with CDC light traps (*20*) from June to October of 2003 and 2004. Traps were placed in 16 areas of the rural environment where the first cases of meningitis by TOSV appeared (*21*) (Figure 1). Sandflies were trapped after dusk until dawn. Traps were immediately transported to the laboratory to pool the individual vectors by sex and trapping area. In the 2003 season, pools of male sandflies were used for taxonomic classification, and pools of female sandflies were tested for TOSV by RT-PCR. In the 2004 season, up to 10% of individual sandflies were separated for taxonomic classification. The remaining insects were pooled by sex and trapping area and tested for TOSV by viral culture and RT-PCR. Taxonomic classification of sandflies was carried out according to Gil-Collado et al. (*19*).

#### Viral Culture

Phlebotomines were introduced in vials with sterile crystal beads and 0.5 mL minimal essential medium supplemented with 20% fetal bovine serum and antimicrobial mix (0.4 mg/mL gentamicin, 0.5 mg/mL vancomycin, and 2.5 μg/mL amphotericin B). Vials were vortexed and centrifuged at 13,000 rpm for 5 min. A 200-μL aliquot of the supernatant was injected into tubes with African green monkey kidney cells; the remaining supernatant was frozen at –80°C. The pellet with the phlebotomines was used for RT-PCR. Tube cultures were incubated at 37°C and examined daily for the appearance of cytopathic effect (CPE). Tubes with positive CPE were tested for TOSV by RT-PCR.

#### RT-PCR for Testing TOSV in Sandfly Pools

Viral RNA from cell cultures was extracted by using QIAamp viral RNA kit (Qiagen, Hilden, Germany), following the manufacturer's instructions. Viral RNA from sandflies was isolated by using the same kit, with minor modifications. Briefly, 500 μL lysis buffer was added to the tubes containing the phlebotomine sandflies, tubes were vortexed and centrifuged at 13,000 rpm for 10 min, and 250 μL of the supernatant was used for RNA extraction. A generic RT-nested-PCR method was used to detect TOSV RNA as described previously (*17*), which amplifies a 244-bp fragment of the L gene of phleboviruses. Sequences of the primers are shown in Table 1.

**Table 1:** Primers used for RT-PCR amplification of the L (partial) gene and the N gene of TOSV*

Primer	Sequence	PCR
NPhlebo1+	5´ _2047_ATGGARGGITTTGTIWSICIICC_2069_ 3´	L gene–1st
NPhlebo1–	5´ _2600_AARTTRCTIGWIGCYTTIARIGTIGC_2575_ 3´	L gene–1st
NPhlebo2+	5´ _2074_WTICCIAAICCIYMSAARATG_2094_ 3´	L gene–2nd
NPhlebo2–	5´ _2318_TCYTCYTTRTTYTTRARRTARCC_2296_ 3´	L gene–2nd
TosS1+	5´ _4_CAGAGATTCCCGTGTATTAAAC_25_ 3´	N gene–1st
TosS1–	5´ _1052_GAGTGCTGCCAAGTCTTATGAC_1031_ 3´	N gene–1st
TosS2+	5´ _4_CAGAGATTCCCGTGTATTAAACAAAAGC_31_ 3´	N gene–2nd
TosS2–	5´ _1004_TAGAGAAACTGCTCTTTCCACC_983_ 3´	N gene–2nd

*RT-PCR, reverse transcription–polymerase chain reaction; TOSV, Toscana virus.

#### Sequencing Reactions on RT-PCR–Positive Pools

Specific TOSV detection was achieved by sequencing PCR products, as described previously (*17*). Briefly, PCR products were purified from a 2% low-melting-point agarose gel with the QIAquick PCR purification kit (Qiagen). Sequencing reactions were performed by using the ABI Prism Big Dye Terminator Cycle Sequencing Ready Reaction Kit (Applied Biosystems, Foster City, CA, USA) and analyzed by an ABI model 377 automated sequencer (Applied Biosystems). Two sequencing reactions were carried out on each PCR product by using the sense and antisense primers of the nested-PCR step. TOSV-specific sequences were confirmed by BLAST (basic local alignment search tool) search against GenBank databases. Sequences of the L gene from positive pools were compared with the corresponding sequence of the Italian strain ISS Phl.3 and with 1 Spanish TOSV isolate (STI) obtained previously (*17*) with the ClustalW Multiple Sequence Alignment program (1.82 version; European Bioinformatics Institute, Cambridge, UK).

### Molecular Characterization of Spanish TOSV

To achieve a better characterization of STIs, the N gene was targeted. Sequences of the N gene of different phleboviruses were identified by BLAST search and aligned. GenBank accession numbers of the sequences were TOSV ISS Phl.3: X53794; Rift Valley fever virus (RVFV): NC 002045; SFSV: J04418; Punta Toro virus: K02736; and Uukuniemi virus (UUKV): NC 005221. Subsequently, different combinations of primers were selected to amplify 1 STI. Finally, the obtained sequence and the 1 from the Italian strain ISS Phl.3 were aligned, and specific primers were redesigned to amplify STIs recovered from sandflies and previous STIs from patients (*17*) (Table 1). RT-PCR conditions are available on request. Sequences were obtained and compared with the Italian strain as described above.

#### Phylogenetic Analysis

Phylogenetic analysis of the sequences from the partial L gene and the complete N gene was carried out with MEGA 3 program (*22*) by using the Kimura 2 parameter model for nucleotides and amino model with Poisson correction for amino acids to calculate distances between sequences with confidence values of 1,000 bootstrapping trials. Phylogenetic trees of the L gene were constructed from TOSV obtained from sandflies and previous STIs and from different phleboviruses. Available GenBank accession numbers of the phleboviruses sequences were TOSV ISS Phl.3: X68414; RVFV: X56464; UUKV: D10759; and phlebovirus Chios-A (Chios): AY293623. Phylogenetic trees of the N gene were constructed from STI sequences obtained from sandflies and patients and from the phleboviruses sequences described above.

### Statistical Analyses

To calculate the sample size for the seroprevalence study, we applied the estimation of proportions model for infinite populations. We made the following assumptions: an estimated seroprevalence of 10% in our area based on previous studies (*7**,**15*), 95% confidence level, and 5% precision level. With these premises, the minimum sample size was 139. Results were statistically analyzed with the SPSS 12.0.1. Program (SPSS, Chicago, IL, USA). Along with descriptive statistics, univariate analysis was conducted on the results obtained from the seroprevalence study by χ^2^ test. A p value <0.05 was considered significant. TOSV infection rate in phlebotomine sandflies by means of RT-PCR was calculated with Pool Screen 2.0 program (*23*).

## Results

### Prevalence Study of Anti-TOSV IgG Antibodies

Anti-TOSV IgG was analyzed in 979 human serum samples. The study population was distributed by geographic area proportional to Granada's population (Table 2). By sex, 472 (48.2%) were males, and 507 (51.8%) were females. By age groups, 183 (18.7%) were <18 years, 662 (67.6%) were aged 18–65 years, and 134 (13.7%) were >65 years (see Materials and Methods).

**Table 2:** Demographic data of Granada population and distribution of the study population by geographic areas

Area	Granada population			Study population, n (%)
	Total, n (%)	Males, n (%)	Females, n (%)	
Urban	240,522 (29.2)	111,774 (46)	128,748 (54)	287 (29.3)
Metropolitan	227,994 (27.6)	114,836 (50)	113,158 (50)	282 (28.8)
South	157,803 (19.1)	78,871 (50)	78,932 (50)	146 (14.9)
West/Southwest	70,397 (8.5)	35,429 (50)	34,968 (50)	72 (7.4)
North/Northeast	128,299 (15.6)	64,214 (50)	64,085 (50)	192 (19.6)
Total	825,015 (100)	405,124 (49)	419,891 (51)	979 (100)

The overall prevalence of anti-TOSV IgG was 24.9% (range 9.4% in persons <15 years to 60.4% in those >65 years, p<0.001) (Table 3). No statistical differences were observed by geographic area or sex. However, when the urban area was compared with rural areas, seroprevalence rate was statistically higher in the latter group (20.6% in the urban area vs 26.7% in the rural areas; p = 0.042).

**Table 3:** Results of prevalence study of anti-TOSV immunoglobulin G, Granada*

Area	<15 y, no. (pos)	15–40 y, no. (pos)	41–65 y, no. (pos)	>65 y, no. (pos)	No.	Pos (%)
Urban	52 (2)	141 (22)	60 (20)	34 (15)	287	59 (20.6)
Metropolitan	51 (7)	127 (24)	62 (18)	42 (28)	282	77 (27.3)
South	34 (3)	76 (14)	54 (12)	28 (19)	192	48 (25.0)
West/southwest	3 (0)	40 (7)	20 (10)	9 (6)	72	23 (31.9)
North/northeast	19 (3)	71 (15)	35 (6)	21 (13)	146	37 (25.3)
Total	159 (15, 9.4%)	455 (82, 18%)	231 (66, 28.6%)	134 (81, 60.4%)	979	244 (24.9)

*TOSV, Toscana virus; pos, positives.

### Vectors for TOSV

For taxonomic classification, 1,431 sandflies were studied, 1,286 males and 145 females. The most abundant species was *P. perniciosus* (68.7%), followed by *Sergentomyia minuta* (16.4%), *P. sergenti* (7.1%), *P. papatasi* (5.7%), and *P. ariasi* (0.5%); 1.6% of the sandflies were classified as *Phlebotomus* spp.

The presence of TOSV was investigated in 103 sandfly pools, 22 pools of females in 2003 and 81 pools in 2004 (42 pools of males and 39 pools of females). Three of the 81 pools obtained in 2004 were positive for TOSV by RT-PCR (2 female and l male pool); 2 were also positive by cell culture. The infection rate for TOSV in phlebotomine sandflies was 0.05% (95% confidence interval 0.1–0.009).

### Molecular Characterization of Spanish TOSV

#### Genetic Analysis of the L Gene

**Figure 2:**
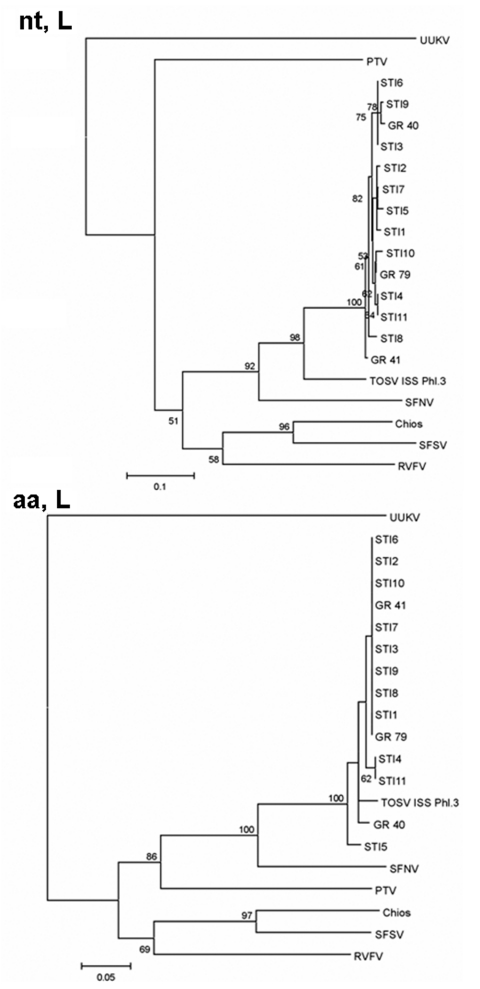
Phylogenetic trees illustrating the relationship between representatives of different phleboviruses and the Spanish Toscana virus (TOSV) within the nucleotide (nt, L) and the deduced amino acid sequences (aa, L) of the L (partial) gene. GR40 and GR41 correspond to TOSV isolates obtained from sand flies. GR79 corresponds to a reverse transcription–polymerase chain reaction–positive pool of sandflies. STI1–STI11 were recovered from patients with aseptic meningitis diagnosed from 1988 to 2002 as described in (*17*). Abbreviations and GenBank accession numbers are indicated in the text. Bootstrapping values >50 are indicated at the nodes.

TOSV sequences of the L (partial) gene from vectors and that from a human STI were almost identical. At the nucleotide level, 19%–20% diversity was observed between sequences obtained from Spanish samples and those of the Italian strain, whereas the homology at the amino acid level was almost 100% (Table 4). Phylogenetic analysis yielded the same results (Figure 2). The phylogenetic tree of nucleotide sequences show 1 group containing all TOSV, in which the Spanish ones form, with a bootstrap value of 100, a different cluster. This diversity is not reflected in the amino acid sequences since both the Spanish and Italian TOSV group into a unique cluster.

**Table 4:** Homology of nucleotide and deduced amino acid sequences of L (partial) gene and complete N gene in Toscana viruses from Spain and the Italian strain ISS Phl.3*

L gene	STI1	ISS Phl.3	GR40	GR41	
nt					
ISS Phl.3	0.199				
GR40	0.010	0.199			
GR41	0.015	0.185	0.026		
GR79	0.010	0.205	0.020	0.015	
aa					
ISS Phl.3	0.015				
GR40	0.015	0. 030			
GR41	0.000	0.015	0.015		
GR79	0.000	0.015	0.015	0.000	
N gene	STI1	STI2	ISS Phl.3	STI6	GR40
nt					
STI2	0.020				
ISS Phl.3	0.116	0.123			
STI6	0.005	0.017	0.118		
GR40	0.015	0.016	0.123	0.012	
GR41	0.020	0.021	0.130	0.017	0.016
aa					
STI2	0.000				
ISS Phl.3	0.000	0.000			
STI6	0.000	0.000	0.000		
GR40	0.000	0.000	0.000	0.000	
GR41	0.000	0.000	0.000	0.000	0.000

*GR40, GR41, and GR79: TOSV obtained from pools of sandflies; STI1, STI2, and STI6: Spanish Toscana virus isolates recovered from patients in previous studies (*17*); ISS Phl.3: Italian TOSV strain (GenBank accession no. X68414 [L gene] and X53794 [N gene]); nt, nucleotides; aa, amino acids.

#### Genetic Analysis of the N Gene

**Figure 3:**
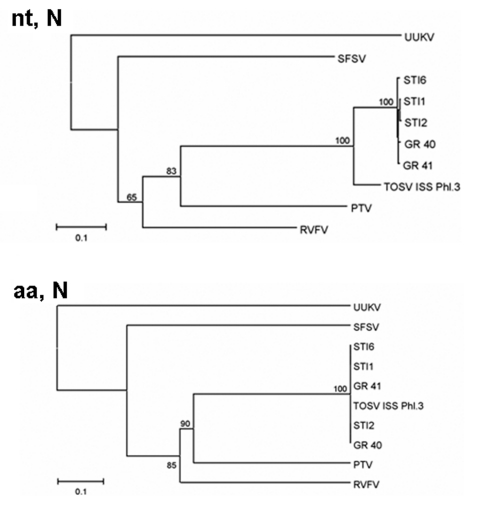
Phylogenetic trees illustrating the relationship between representatives of different phleboviruses and the Spanish Toscana virus isolates within the nucleotide (nt, N) and deduced amino acids sequences (aa, N) of the N gene.

Nucleotide and amino acid sequence homology within the N gene was ≈98%–100% among STIs from vectors and patients. Compared with the Italian strain, a 100% homology at the amino acid level was observed between STIs and the Italian strain. However, nucleotide sequences showed a 12%–13% difference between the strains (Table 4). The phylogenetic tree of nucleotide sequences shows that, within the TOSV group, STIs form a different cluster. However, at the amino acid level, both STIs and Italian TOSV strain group into a unique cluster (Figure 3).

## Discussion

The overall 25% seroprevalence rate found in this study is similar to the rates reported in Mediterranean areas (*14**,**24*). Other seroprevalence studies carried out in northern Europe report lower rates (*13*), which demonstrates that TOSV infection is endemic in Mediterranean countries. The high seroprevalence rates suggest that the diagnosis of TOSV infection is frequently missed since most cases are mild, as occurs with SFSV and SFNV infections (*6*), a severe illness involving the central nervous system develops in only a few patients. The increasing seroprevalence rates concurrent with age demonstrate that the Granada population is exposed to TOSV throughout life. Thus, a study on outpatients attending primary care services is necessary to assess the role of TOSV in human disease.

TOSV was first isolated from *P. perniciosus* in Italy (*8*). Although, the sandflies used in this study for detecting TOSV were not used for taxonomic classification, ≈70% of captured insects were *P. perniciosus*, which suggests that this species is the main vector for TOSV in our area. Furthermore, *P. perniciosus* accounted for 50%–90% of the sandflies captured in the area where TOSV-positive pools were detected. The rest were classified as *S. minuta*, *P. sergenti*, and *P. papatasi*, none of which are known vectors for TOSV.

TOSV was detected in 3 of 103 pools. By using the Pool Screen 2.0 program (*23*), an infection rate of 0.05% was obtained in the sandflies, which is much lower than the infection rate of 0.2% reported in Italy (*25*), where more cases of TOSV infection have been recorded (*11**,**26*). The fact that the seroprevalence rate is similar to the rates reported in Italy, another endemic area, and that the virus infection rate in sandflies is much lower, could be due to increased exposure to the vector, as occurs in our area, which is mainly rural.

To investigate the genetic relationship of TOSV detected in sandfly pools with the Italian strain and STIs recovered previously, sequence analysis of a fragment of the L gene and the complete N gene was performed. Similar results were obtained with both regions of the genome. Nucleotide and amino acid sequence homology of TOSV from vectors and patients was ≈100%. The differences between TOSV (from vectors and patients) and the Italian strain within the nucleotide sequences indicate that at least 2 lineages of TOSV, Italian and Spanish, are circulating. These changes are synonymous because almost identical amino acid sequences were found in all analyzed TOSV. This finding suggests that, at least within these regions of TOSV genome, constraints against amino acid changes exist. This fact has already been described with RVFV isolates from different areas (*27*).

Despite the high seroprevalence rates found in this study, fewer cases of severe disease caused by TOSV occur in our area (*21*) than in other countries (*11*). A possible explanation for this could be that Spanish TOSV are less neurovirulent than the Italian. Whether changes in other parts of the genome, such as the noncoding regions, affect the neurovirulence of this virus, as described for tickborne encephalitis virus (*28*), needs to be investigated.

The finding of this new lineage of TOSV was not an isolated event. Nucleotide sequences of TOSV from sandflies collected during 2003–2004 were almost identical to those obtained from patients from 1988 to 2002. The genetic diversity between STIs and the Italian strain could be partially explained by vector characteristics. Two lineages of *P. perniciosus* have been reported (*29*), the typical lineage found in Morocco, Tunisia, Malta, and Italy, and the Iberian lineage. These lineages remain isolated because sandflies move in short hops, flying no more than a few hundred meters from their resting places.

As occurs with WNV, for which several lineages have been reported, the last in central Europe (*30*), more lineages of TOSV may circulate in other areas. To assess this possibility, further investigation would be necessary. Moreover, sequencing of the S segment of a TOSV isolate recently described in southern France showed 87% and 100% homology with the reference strain within nucleotide and peptide sequences, respectively (*31*). Although GenBank accession for this sequence is not yet available, this isolate could belong to the Spanish TOSV lineage since differences between this isolate and the Italian strain are similar to the differences that we found in our isolates.

In conclusion, the study and surveillance of arbovirus infections should be considered worldwide since they may cause emergent diseases, many of which may be life-threatening. One part of this study should focus on the vectors and host spectrum of these viruses to control transmission to humans.
